# Illicit substances identified in the urine of 11.170 suspected drug users in North Tunisia

**DOI:** 10.11604/pamj.2021.38.20.26781

**Published:** 2021-01-11

**Authors:** Nadia Chaouali, Bilel Moslah, Kamel Ben Salem, Dorra Amira, Abderazzek Hedhili, Nabil Ben Salah

**Affiliations:** 1 Toxicology and Environmental Research Laboratory LR12SP07, Center for Urgent Medical Assistance, Montfleury 1008 Tunis Cedex, Tunisia,; 2 Faculty of Pharmacy, University of Monastir, Monastir, Tunisia,; 3 Faculty of Medicine of Sousse, Sousse, Tunisia,; 4 Intensive Care Unit, Center for Urgent Medical Assistance, Montfleury 1008 Tunis Cedex, Tunisia

**Keywords:** Prevalence, cannabis, cocaine, urinalysis, Tunisia

## Abstract

**Introduction:**

according to the latest World Drug Report, 271 million people worldwide (5.5% of the global population) aged 15-64 years are drug users. Drug addiction and trafficking became an urgent public health problem that affects human health and social life. A cross-sectional study was conducted from January 2016 to December 2018, to identify the socio-demographic profile of drug users captured by the anti-narcotics squad in North of Tunisia (North African country) and to type, through toxicological analysis, the nature of the substances consumed.

**Methods:**

data were collected from expertise files of 11170 suspected drug users. Fresh urine samples were collected from suspected drugs users and submitted in the toxicology laboratory of the center for Urgent Medical Assistance (Tunis) for forensic urinalysis. Drugs screening was carried out by immunochemical methods. Positive samples were analyzed with gas chromatography coupled to mass selective detector (GC-MS) for confirmation.

**Results:**

the investigation revealed that drug users were mainly males 97.4% (sex ratio 37), the median age was 29 ± 7.91 years, 91.3% were singles and 79% were daily workers. On a total of 11170 urine samples screened, 5 409 (48.4%) were positives for illicit drugs. The prevalence of positive samples was 55.4% in 2016; 50.45% in 2017 and 40.8% in 2018. Cannabis was the most widely consumed drug (95%), followed by Benzodiazepines (1.2%), Buprenorphine (1%), cocaine (0.95%), MDMA (0.24%) and opiates (0.13%). Polydrug abuse was observed in 79 specimens (1.5%).

**Conclusion:**

this study provides an overview of the illicit drug consumption in the north of Tunisia (knowing that nowadays epidemiological data are almost same since 2016) in order to set up an effective policy to fight against drugs and addictive behaviors and to provide health professionals with the epidemiological elements necessary for better medical care of drug users.

## Introduction

Drug use became an urgent public health problem that affects human health, social life and economy worldwide. According to the latest *World Drug Report*, 271 million people worldwide (5.5% of the global population) aged 15-64 years are drug users, which representing one in every 18 people [1]. In Tunisia (North African country), traffic and consumption of psychoactive substances have risen substantially since the uprising of 2011. Drugs of abuse became increasingly available particularly to school students [2]. Indeed, teens access illicit drugs in schools, from street dealers and through internet purchase « Darknet » [3]. Previous epidemiological studies confirmed the increase of drug abuse in North Africa. An epidemiologic study conducted on a total of 28 298 cases in Tunisia from 2010 to 2015, stated that cannabis was the most widely consumed illicit drug among young adults followed by benzodiazepines, buprenorphine, cocaine and MDMA (Ecstasy) [4]. According to the Mediterranean School Survey Project on Alcohol and Other Drugs (MedSPAD II) conducted in Tunisia in 2017 [5]; tobacco, alcohol and cannabis are the substances which secondary school students use most frequently. The frequency of consumption was (25.1%) for tobacco, (6.3%) for alcohol, (3.8%) for cannabis, (3.8%) for glue, (3%) for psychotropic drugs, (1.4%) for Ecstasy and under (1.0%) for cocaine and Buprenorphine. These frequencies have increased substantially compared to the first MedSPAD survey conducted in 2013 [2], whereas these frequencies still less than those noted in other Mediterranean countries (Cyprus, France, Greece, Italy, Portugal, Malta, Lebanon and Morocco) [5]. An epidemiologic investigation was conducted to evaluate the prevalence of drug abuse in 11170 suspected drug users by analyzing urine samples collected from January 2016 to December 2018.

## Methods

It was a cross-sectional study. The investigation was conducted in the toxicology laboratory of *the center for Urgent Medical Assistance* which is the public reference center for forensic detection of drug abuse. We have extracted the variables of interest (sex, age, occupation, place of residence and marital status) of each individual tested from expertise files using a data collection sheet. These data were registered by police enforcement in expertise files which exist in paper format and were stored in the laboratory archives.

### Biological samples

In Tunisia, drug testing in biological samples is carried out at the request of the judicial authority. Individuals suspected of drug use (or deal) were arrested by the anti-narcotics squad and transferred to the national forensic laboratory for drug urinalysis. Urine samples were collected from each individual by a police officer with the suspect's consent. Only one sample was taken from each individual. A serial number was given to each sample and the urine analysis was carried out anonymously. Thirty (30) ml of fresh urine samples were collected in a clean and dry polyethylene container, tightly wax sealed then immediately stored at +4°C until forensic analysis. The urinalysis was conducted within 24 to 48 hours then samples were stored at -20°C. Diluted specimens were excluded from this study. During the period lasting from January 2016 to December 2018; 11170 samples were tested as follows: 3610 samples in 2016; 3368 in 2017 and 4192 in 2018.

### Toxicological analysis

Each specimen was preliminary screened with a urine drug test strips (Acro rapid Test®, AcroBiotech, Inc USA) for numerous classes of illicit substances (cannabinoides, cocaine, MDMA benzodizepines, buprenorphine, barbiturates, and opiates). Urine specimens exhibiting visible precipitates were centrifuged to obtain a clear supernatant for testing. It was about a rapid immunochromatographic assay. The strip was immersed in the sample for 5 seconds; urine was drawn along the rapid test by capillary action during 5 minutes, if two red lines appear next to both the control region and test region, the test was negative. If a line appears only next to the control zone, the test was positive. Positive specimens (for one or more compounds) were tested by immunochemical methods (fluorescence polarization immunoassay methods (FPIA)/ enzyme-multiplied immunoassay technique EMIT), using the Cobas Integra 400® analyser (Prochidia Laboratories. Roche, Switzerland). Subsequent positive samples were confirmed by gas chromatography coupled to mass selective detector (GC-MS).The analytical technique of (GC-MS) combines the efficient separating power of gas chromatography with the high sensitivity and specificity of mass spectrometric detection. This method was the most conclusive method of confirming the presence of drugs in urine [6]. The analysis was carried out on an Agilent 6890 Gas Chromatograph interfaced to a model HP 5973 Mass Selective Detector. The identification of the eight compounds was carried out using their retention times and their mass spectra (Agilent commercial libraries).

### Data analysis

The variables of interest were listed on a SPSS table; variables such as sex, occupation, and marital status were coded. The data analysis plan was based on descriptive statistics (positive samples frequency, the median age of drug users, the sex ratio and the percentage of each class of illicit substances) were processed by SPSS Statistics 24.0.

## Results

### Drug positive urines samples

**Figure 1:**
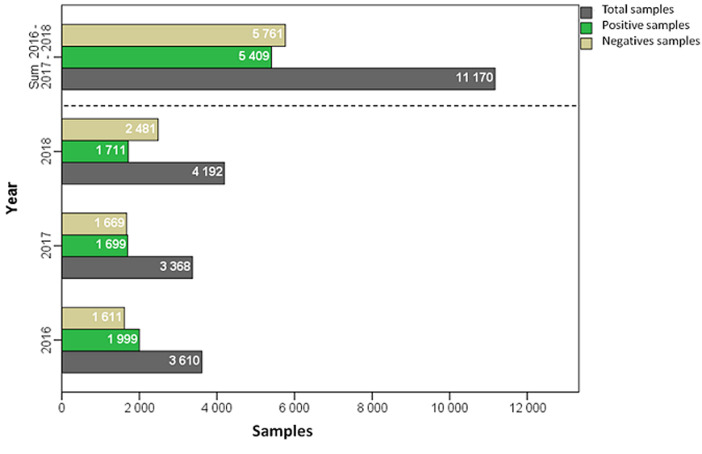
prevalence of drug users from 2016 to 2018

**Figure 2:**
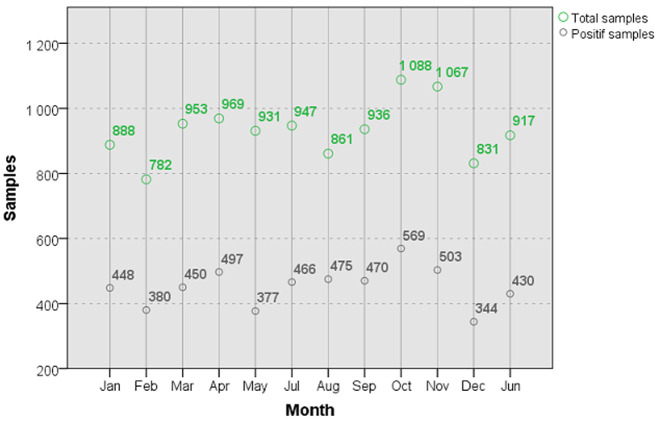
monthly distribution of the average positive samples compared to total tested samples during 2016-2017-2018

A compilation of three years (2016 to 2018) of epidemiologic investigation was conducted. Summary, 11170 arrested people suspected to be drug consumers was reported. Out of specimens tested 5409 samples (48.4%) were positives for one or more classes of drugs of abuse. We reported 1999 positive cases in 2016; 1699 in 2017 and 1711 in 2018. Figure 1 summarizes the number of positive and negative samples listed during three years. We found that between 782 and 1088 arrests are made each month from 2016 to 2018, of which 50% are positive for drugs of abuse (450 ± 60 samples/ month). Figure 2 illustrates the distribution of total arrests by month and positive rates of drug users (average of three years 2016-2017-2018).

### Socio-demographic characteristics of drug users

The current investigation showed that drug users were mainly males 97.4% (sex ratio 37). All age ranges (from 8 to 88 years old) were affected by drug use; the median age was 29 ± 7.91years, 65.4% were less than 30 years old and 91.3% were singles. Socio-demographic characteristics of drug users screened positive in the current study are summarized in Table 1.

**Table 1:** demographic characteristics of drug users

Variables	Total n (%)	2016 n	2017 n	2018 n
**Sex**				
Males	5267 (97.4)	1963	1649	1655
Females	142 (2.6)	36	50	56
**Ages (years): Mean(±SD)**				
Males	27 (±8.1)	27(±7.8)	27(±8.5)	27(±8.9)
Females	24 (±8.9)	27(±7.4)	25(±6.3)	26(±7.8)
**Marital status**				
Single	4940(91.3)	1869	1573	1498
Married	423(7.9)	120	109	194
Divorced	45(0.8)	9	17	19
**Occupation**				
Daily Worker	4276(79)	1626	1391	1259
Students	402(7.45)	188	113	101
Unemployed	146(2.7)	61	46	39
Military	93(1.7)	42	37	14
Others	407(7.52)	54	80	273

### Prevalence of illicit drugs

**Figure 3:**
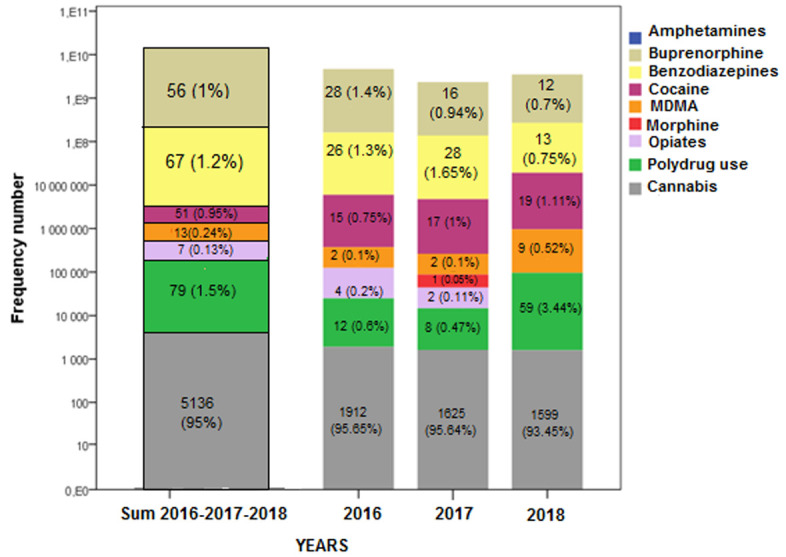
positivity rates of each class of illicit drugs tested for 2016, 2017 and 2018

Positive urine samples revealed the presence of several classes of illicit drugs. Substances detected were cannabinoides, Benzodizepines, Buprenorphine, cocaine, MDMA and opiates. Barbiturates were not detected. Over the three years of study, we have noticed that Cannabis was the most widely consumed illicit drug (95%), followed by Benzodiazepines (1.2%), Buprenorphine (1%), cocaine (0.95%), MDMA (0.24%) and opiates (0.13%). Polydrug use was observed in 79 specimens (1.5%). Figure 3 illustrates in detail the positivity rates of illicit drugs detected all over the 3 years of investigation (2016, 2017 and 2018).

## Discussion

The current study showed that the prevalence of positive samples was directly proportional to the number of total samples tested every year, approximately 50% of suspected drug consumers trapped by narcotic squad every year were positive for targeted drugs (1803 ± 138 individuals per year). We also noted that the rate of positive cases was stable over the months (450 positive samples ± 60 per month). Nevertheless, a decrease in drug positive cases has been observed as follows; 55.4% in 2016; 50.45 % in 2017 and 40.8% in 2018, these rates are in contrast with the large quantity of narcotics seized every month in the region. We suggest that this contrast may be due to the emergence of NPS (new-generation synthetic psychoactive substances) which are growing worldwide; beginning with Europe, followed by Asia, Africa, the Americas, and Oceania [7]. The consumption of designer drugs is underestimated because this new class of narcotics (synthetic cannabinoids, cathinones derivatives, Alpha-PVP and mephedrone) is undetectable in biological samples by standard toxicology screens witch lead to false negative results. In many countries, this issue is still relevant because of the lack of analytical methods and the unavailability of standards [8]. However, future testing will need to be performing to confirm this statement.

### Epidemiologic characteristics of drug users

More male drug users were observed compared to female (97.4% vs 2.6%). The existing research points to unequal opportunities (also relating to social and cultural norms) in access to illicit drugs as one of the reasons for differences in the prevalence of drug use between men and women [9]. According to the French Monitoring Center for Drugs and Drug Addiction (OFDT), men and women do not have the same behavior towards drugs [10]. Curiosity and induction by a partner are the most common reasons of initiation drug use for women [11]. Drug abuse concerns all generations but maximum prevalence was observed for adults aged 18 to 34 years (77%). According to the 2004 Canadian Addiction Survey, almost 61.4% of youths used cannabis in their lifetime [12]. In Europe, 12.1% of people aged from 15 to 34 state that they have consumed cannabis in the past year, versus 16.7% in France and 24.1% for the United States [13]. According to consumers, drugs use is an expression of persons in search for experience, identification and pleasure [14, 15]. In the other hand, drug use appears to be highly dependent on the individuals´ professional status; maximum prevalence was observed among daily workers (79%); who suffer from professional instability, poverty and have lower educational level. According to several studies, the use of drugs is mainly linked to factors such as poverty, unemployment, lower social status, instability and violence in families and communities [16].

### Prevalence of illicit substances consumed by drug users from 2016 to 2018

Cannabis was detected in 5136 (95%) of the urine samples tested, it was the most widely consumed illicit substance, probably because of it relatively low cost and easy availability compared to other drugs [17]. Besides, the legislation is much less severe when it comes to cannabis than with hard drugs. Moreover, important cannabis trafficking from neighboring countries existed in the region. According to the report of the International Narcotics Control Board (INCB), in 2017 the largest quantities of cannabis were seized in Africa [18]. Cannabis trafficking follows a circuit that goes from North Africa to Europe across Spain [19]. In the world, Cannabis continues to be the most widely used drug; in 2017, 188 million (3.8% of global population) people are cannabis users [1]. Over 24 million people in USA aged 18 (or over) were estimated to be users of cannabis [20]. The current results showed that 79 specimens (1.5%) were positive for more than one type of psychoactive substances, so-called polydrug use. This misuse increased significantly since 2016 (0.6% in 2016 vs 3.44% in 2018), the most observed combination was cannabis with benzodiazepines (97%). The polydrug use is remains a serious public health problem that affects people around the world; in USA, simultaneous co-use of alcohol and marijuana was the most common combination observed (83%) followed by crack and heroine (56%) [21]; According to Ludici et al, drug users mix two or more illicit drugs in combination to intensify their effects and make the experience stronger and more euphoric, but consequences are even more harmful [22]. On the other hand, Benzodiazepines (BZD) were detected in 67 urine samples (1.2%). These psychotropic pills have a powerful addictive potential but remains much less dangerous than other drugs if not associated with alcoholic beverages [23]. BZD are the third most commonly misused molecules in the USA and European countries (approximately 2.2% of the population) [24]. Buprenorphine (BUP) was detected in 56 samples (1%), but consumption was gradually decreased over the years. Some addicts use BUP intravenously because it provides the same euphoric effects as opiates but at a lower cost [25]. It should be noted that BUP is the most commonly injected drug in Tunisia. Numerous studies have documented this issue: In France, buprenorphine replaces heroin as the mainly injected product. The Misuse by injection would affect, according to AFSSAPS, about 15% of patients in treatment [26]. In Europe, Georgia and Finland face high prevalence of injection of this molecule [27]. Sample tested positives for cocaine and MDMA/Ecstasy, was markedly lower compared with cannabis, but would tend to increase from 0.75% in 2016 to 1.1% in 2018 for cocaine and from 0.1% in 2016 to 0.52% in 2018 for MDMA. This upward trend is in part due to the expansion of the global market of amphetamine and cocaine, according to the United Nations Office on Drugs and Crime (UNODC), the seizures of amphetamines across the world quadrupled between 2009 and 2018 while the global quantity of cocaine seized in 2017 was 1275 tons, the largest quantity ever reported. In addition, the global illicit manufacture of cocaine reached a record amount of 1976 tons in 2017 (an increase of 25 % on the previous year) [28]. Otherwise, access to opiates such as (morphine, Heroin) was minor (0.1%), we suggest that it is because of the high cost and the unavailability of opiates in the region. According to similar studies, we noticed that drug use rates was different from one country to another; in Greece, cannabis was detected in (84%) of the samples, followed by cocaine and MDMA (26%) and (32%) of samples respectively [29]. In Italy, from 2010 to 2014, cannabis and cocaine were the illicit drugs most used (2014 prevalence 3% and 0.43% respectively) followed by opioids (0.17%) and amphetamines (0.14%) [30]. In North Western Nigeria, the most used drug among youths in a rural agrarian community was Tramadol (52.8%) and Marijuana [31]. A population-based survey in South Africa revealed that cannabis use was 4.0%, followed by sedatives or sleeping pills 0.4%, amphetamine-type stimulants 0.3%, cocaine 0.3%, opiates 0.3%, inhalants 0.2% and hallucinogens 0.1% [32].

### Limitations

The main limitations of this study were firstly, the short detection window of drugs in urine samples; it would be suitable to use an alternative biological matrix (such as hair samples) to avoid false negative results. Secondly, the inability to detect new-generation synthetic psychoactive substances by standard methods in biological samples; new methods must be developed using ultra-performance liquid chromatography tandem mass spectrometry (LC-MS/MS).

## Conclusion

The current study revealed that the typical profile of drug user has changed; it was no longer a single young man delinquent, unemployed, with low educational level and resident in an urban area; presently, drugs of abuse are accessible to all social classes from all age groups, residing in urban or rural localities. Cannabis was the most common used drug; cocaine and MDMA are expanding as everywhere in the world. This study provides an overview of the illicit drug consumption in the north of Tunisia (knowing that nowadays epidemiological data are almost same since 2016) in order to set up an effective policy to fight against drugs and addictive behaviors and to provide health professionals with the epidemiological elements necessary for better medical care of drug users.

### What is known about this topic

- Drug use is infrequent and only affects young people and offenders;
- Cannabis is the most used drug.

### What this study adds

- This study has shown that drugs of abuse are accessible to all social classes from all age groups, residing in urban or rural localities;
- The use of cocaine and MDMA has become very common;
- The polydrug use is a new scourge against which we should fight.
